# Super-enhancer-associated long noncoding RNA AC005592.2 promotes tumor progression by regulating OLFM4 in colorectal cancer

**DOI:** 10.1186/s12885-021-07900-x

**Published:** 2021-02-23

**Authors:** Linping Yan, Huanhuan Chen, Li Tang, Pan Jiang, Feng Yan

**Affiliations:** Department of Clinical Laboratory, The Affiliated Cancer Hospital of Nanjing Medical University & Jiangsu Cancer Hospital & Jiangsu Institute of Cancer Research, Baiziting No. 42, Nanjing, 210009 China

**Keywords:** Super-enhancer-associated long noncoding RNA, AC005592.2, Colorectal cancer, Olfactomedin 4

## Abstract

**Background:**

Super-enhancer-associated long noncoding RNAs (SE-lncRNAs) have been reported to play essential roles in tumorigenesis, but the fundamental mechanism of SE-lncRNAs in colorectal cancer (CRC) remains largely unknown.

**Methods:**

A microarray was performed to identify the differentially expressed SE-lncRNAs between CRC tissues and peritumoral tissues. A novel SE-lncRNA, AC005592.2, was selected from these differentially expressed SE-lncRNAs to explore its effects on CRC development. Fluorescence quantitative real-time PCR (qRT-PCR) was used to assay the expression of AC005592.2 in CRC tissues and cell lines. Functional assays were applied to identify the biological effects of AC005592.2 in CRC cells. Furthermore, RNA-seq was employed to predict potential targets of AC005592.2.

**Results:**

AC005592.2 was significantly increased in CRC tissues and cells. High expression of AC005592.2 was significantly associated with TNM stage and tumor differentiation in CRC patients. Knockdown of AC005592.2 suppressed CRC cell proliferation, invasion and migration but promoted apoptosis, while AC005592.2 overexpression exerted the opposite effects on CRC cells. In addition, AC005592.2 positively regulated the expression of olfactomedin 4 (OLFM4), which was also upregulated in CRC tissues.

**Conclusion:**

The findings suggested that AC005592.2 is a crucial promoter of CRC progression and may serve as an attractive therapeutic target for CRC.

**Supplementary Information:**

The online version contains supplementary material available at 10.1186/s12885-021-07900-x.

## Background

Colorectal cancer (CRC) is one of the most common malignant tumors. According to global cancer statistics in 2018, CRC ranks as the third cause of cancer-related morbidity (10.2%) and the second leading cause of cancer-related mortality (9.2%) worldwide. The progression of CRC is closely related to the mutation of oncogenes and tumor suppressor genes [1], and extensive research has been carried out in this field. However, there are still numerous oncogenes and tumor suppressor genes that have not yet been studied, and their role in CRC is completely unknown. Therefore, further deciphering of the mechanism of some unknown CRC-related genes may provide us with more effective therapeutic strategies to improve the overall survival rate of CRC patients.

Super-enhancers (SEs) are clusters of enhancers enriched with genomic regulatory elements [2, 3]. In multiple types of mammalian cells, SEs are closely related to essential lineage-specific genes that can be used to regulate gene expression and confirm cell-type specificity by increasing gene transcription over vast genomic distances [2, 4]. Moreover, SEs can regulate the expression of oncogenes and other transcripts important for tumor pathogenesis [5, 6]. Super-enhancer-associated lncRNAs long noncoding RNAs (SE-lncRNAs) are a specific set of lncRNAs transcribed from SE genomic regions. Recent studies have revealed that SE-lncRNAs are usually master RNA regulators in diverse gene expression programs and activate gene expression by transcription factor trapping, chromatin looping, chromatin modification, PolII loading, and release of transcriptional repressors [7–11]. SE-lncRNAs are intimately involved in regulating tumorigenesis [11, 12]. For example, CCAT1-L positively regulates MYC expression by mediating chromatin looping between the MYC promoter and its enhancers to promote CRC progression [11].

In this study, the differentially expressed SE-lncRNAs in four pairs of CRC tissues and peritumoral tissues were analyzed by using a human SE-lncRNA microarray, and a novel CRC-associated SE-lncRNA named ACC005592.2 was identified. The upregulation of ACC005592.2 was significantly correlated with TNM stage and tumor differentiation of CRC patients. Further studies found that ACC005592.2 plays an oncogenic role in CRC progression by promoting cell proliferation, migration, and invasion and restricting apoptosis. Mechanistic research showed that ACC005592.2 might exert its oncogenic actions by regulating olfactomedin 4 (OLMF4). Moreover, SE-lncRNA AC005592.2 has not been reported to regulate OLFM4 expression during CRC progression in any other experimental model.

## Methods

### Clinical samples

A total of 33 pairs of CRC tissues and peritumoral tissues were obtained from patients who underwent surgical resection at the Affiliated Cancer Hospital of Nanjing Medical University (Nanjing, China). None of these patients underwent radiotherapy,preoperative chemotherapy or other tumor-specific therapies. All fresh tissues were stored in − 80 °C until use.

### Arraystar human SE-LncRNA microarray

The Arraystar human SE-lncRNA microarray is used for global profiling of SE-lncRNAs and protein-coding mRNAs and includes approximately 7753 SE-lncRNAs and 7040 coding mRNAs. The microarray analysis was performed by Kangcheng Biology Engineering (Shanghai, China) following the Arraystar standard protocol. Briefly, four CRC tissues and peritumoral tissues were selected to profile the expression of SE-lncRNAs. The dysregulation of SE-lncRNAs was identified and analyzed according to the criteria of fold change > 2 and *P*-value < 0.05. The raw data have been uploaded to the National Center for Biotechnology Information (NCBI) Gene Expression Omnibus (GEO) database (http://www.ncbi.nlm.nih.gov/geo/). The GEO accession number is GSE15102.

### Cell culture

The CRC cell lines HCT-116 (RRID: CVCL_0291), SW480 (RRID: CVCL_0546), SW620 (RRID: CVCL_0547), HCT-8 (CVCL_2515), HT-29 (RRID: CVCL_0320), LoVo (RRID: CVCL_0399), and HCT-15 (RRID: CVCL_0292) and the normal human colon epithelial cell line (FHC, RRID: CVCL_3688) were purchased from the American Type Culture Collection (ATCC). These cells were cultured in 90% Dulbecco’s modified Eagle’s medium (DMEM, Gibco, USA) supplemented with 10% heat-inactivated fetal bovine serum (FBS, Gibco) and 1% penicillin/streptomycin (Invitrogen, USA) in a 37 °C incubator containing 5% CO_2_.

### RNA extractionand reverse transcription

Total RNA was extracted from tissues and cells with TRIzol reagent (Qiagen, USA). The RNA quantity and quality were assessed by a NanoDrop ND-2000 spectrophotometer (Thermo, USA). The integrity of RNA was confirmed by 1% agarose gel electrophoresis. Then, RNA was reverse transcribed to cDNA using Prime-Script RT reagent with gDNA Eraser (TaKaRa, Japan) according to the manufacturer’s instructions.

### Fluorescence quantitative real-time PCR analysis

Fluorescence quantitative real-time PCR (qRT-PCR) analysis was performed using the SYBR Green Master Mix kit (TaKaRa, Japan) on a Life Technologies QuantStudio 6 Flex system (Applied Biosystems, USA) with the following conditions: 95 °C for 10 min and 40 cycles of 95 °C for 15 s and 60 °C for 60 s. The relative mRNA expression was calculated by the 2^-ΔΔCT^ method, and glyceraldehyde 3-phosphate dehydrogenase (GAPDH) was used as an internal control for the CT value. The primer sequences are shown in Supplementary Table S1.

### Protein-coding potential

The protein-coding potential of the AC005592.2 isoform (ENST00000510311.1,ENSG000002231185.2) was assessed by using the Coding Potential Assessment Tool (CPAT, http://lilab.research.bcm.edu/cpat/), Coding Potential Calculator (CPC, http://cpc.cbi.pku.edu.cn/) [13] and PhyloCSF [14, 15]. Here, the UCSC genome browser may serve as an alternative to viewing PhyloCSF scores for AC005592.2 by copying the URL.

### Subcellular localization analysis

The separation of nuclear and cytoplasmic fractions was performed with a PARIS™ kit (Invitrogen, USA) according to the manufacturer’s instructions. Then, the mRNA expression of AC005592.2 in the nucleus and cytoplasm was tested by qRT-PCR. CT values of AC005592.2 were compared to those of GAPDH in the cytoplasm and to U6 in the nucleus.

### siRNA transfection

Three siRNAs targeting AC005592.2 (siRNA-78 sense strand: GGAAGCUAGUAGAAGAUUUTT and antisense strand: AAAUCUUCUACUAGCUUCCTT; siRNA-273 sense strand: GAAUGGCACUUUGGACAAUTT and antisense strand: AUUGUCCAAAGUGCCAUUCTT; siRNA-402 sense strand: GGAGUAGGCUGACCAGUUATT and antisense strand: UAACUGGUCAGCCUACUCCTT) and scrambled negative control siRNA (siRNA-NC, siRNA-NC sense strand: UUCUCCGAACGUGUCACGUTT and antisense strand: ACGUGAGCACUUCGGAGAATT) were synthesized and purchased from GenePharma (Shanghai, China). The Lipofectamine RNAiMAX kit (Invitrogen, USA) was used to transfect siRNA into CRC cells according to the manufacturer’s instructions. Two of the three siRNA sequences were selected for further studies based on the knockdown efficiency, as confirmed by qRT-PCR.

### Construction and infection of vectors for AC005592.2-overexpressing lentivirus

The vectors for AC005592.2-overexpressing lentiviruses and the negative control were designated LV5-AC005592.2 and LV5-NC and constructed by GenePharma (Shanghai, China). CRC cells were infected with LV5-AC005592.2 and LV5-NC in the presence of 5 μg/mL polybrene. After 24 h, the supernatant was replaced with fresh culture medium and then cultured for 48–72 h. The expression of AC005592.2-infected cells was validated by qRT-PCR.

### CCK-8 assay

Cell proliferation was examined with the CCK-8 detection kit (Dojindo, Japan) according to the manufacturer’s protocol. Briefly, CRC cells with different treatments were replantedreplated in 96-well plates at a density of 5 × 10^3^ cells/well and then incubated with 10 μl of CCK-8 solution for 37 °C for 2 h. The proliferation index was measured every 24 h to 96 h at 450 nm absorbance.

### Transwell assay

Cell migration and invasion assays were performed using Falcon Cell Culture Insert (BD Biosciences, USA), and the 8.0 μm pore polycarbonate membranes of invasion assays were coated with Matrigel (BD Biosciences, USA). Briefly, approximately 4 × 10^4^ cells with different treatments were seeded into the upper chamber with 0.2 mL of serum-free DMEM, and0.6 mL of DMEM containing 10% FBS was added to the lower chamber as a chemoattractant. After further incubation for 24–48 h, CRC cells that penetrated the other side of the membrane were fixed with 4% paraformaldehyde and stained with 0.1% crystal violet. The number of cells was counted under a light microscope to determine the cell migratory or invasive ability.

### Cell apoptosis analysis

Cell apoptosis was assessed using the Alexa Fluor 488 Annexin V/Dead Cell Apoptosis Kit (Multi Sciences, China) according to the manufacturer’s instructions. Briefly, CRC cells were harvested and washed in cold phosphate-buffered saline (PBS). The washed cells were centrifuged and resuspended using 1X Annexin-binding buffer to obtain a cell density of 1 × 10^6^ cells/ml. Then,Alexa Fluor 488 Annexin V and 100 μg/mL PI working solution were added to the cell suspension. After incubation at room temperature for 15 min, 1X Annexin-binding buffer was added. Finally, the stained cells were analyzed by flow cytometry.

### Western blot analysis

CRC cells were collected in Radio-immunoprecipitation Assay (RIPA) Lysis Buffer (Biovision, USA) to extract cellular protein, and the protein concentration was detected with a BCA protein assay kit (Thermo, USA). Total lysates were subjected to 10% sodium dodecyl sulfate-polyacrylamide gel electrophoresis (SDS-PAGE) and transferred onto PVDF membranes (Millipore, USA). The membranes were blocked in 5% nonfat dry milk for 1 h at room temperature and incubated with 1:1000 human olfactomedin-4 (OLFM4) antibody (Affinity Biosciences, USA, RRID: AB_2846459) or 1:5000 GAPDH antibody (Abcam, USA, RRID: AB_2049706) at 4 °C overnight. After incubation with goat anti-rabbit IgG H&L (HRP) secondary antibody (Abcam, USA, RRID: AB_955417) for 1 h at room temperature, the protein bands were detected by the ECL method (Millipore, USA).

### RNA sequencing array and bioinformatics analysis

Three siRNA ACC00552.2 transfected HT-29 cells and three negative controls were selected for RNA sequencing (RNA-seq) to identify downstream target genes of AC005592.2. Whole RNA-seq was performed by Guangzhou RiboBio (Guangzhou, China) using the Illumina-NaHiSeq 3000 platform. All the differentially expressed genes (fold change > 2, *P*-value < 0.05) were used for hierarchical clustering, volcano plots, and Gene Ontology (GO) and Kyoto Encyclopedia of Genes and Genomes (KEGG) enrichment analyses. A *P*-value < 0.05 was considered the threshold to define significant enrichment of the genes in the GO and KEGG enrichment analysis.

### Statistical analysis

Statistical analyses were performed by GraphPad Prism v6.0 (GraphPad Software, Inc., La Jolla, CA, USA) and SPSS Statistics Version 20.0 (SPSS, Chicago, IL, USA). The unpaired 2-tailed Student’s *t*-test was used to evaluate significant differences between two groups. Statistical differences for more than two groups were determined by two-way ANOVA and multiple t-tests. All data are presented as the mean ± SD of triplicate independent measurements. A value of *P* < 0.05 was considered to indicate a significant difference.

## Results

### SE-lncRNA and mRNA expression profiles in CRC

**Fig. 1:**
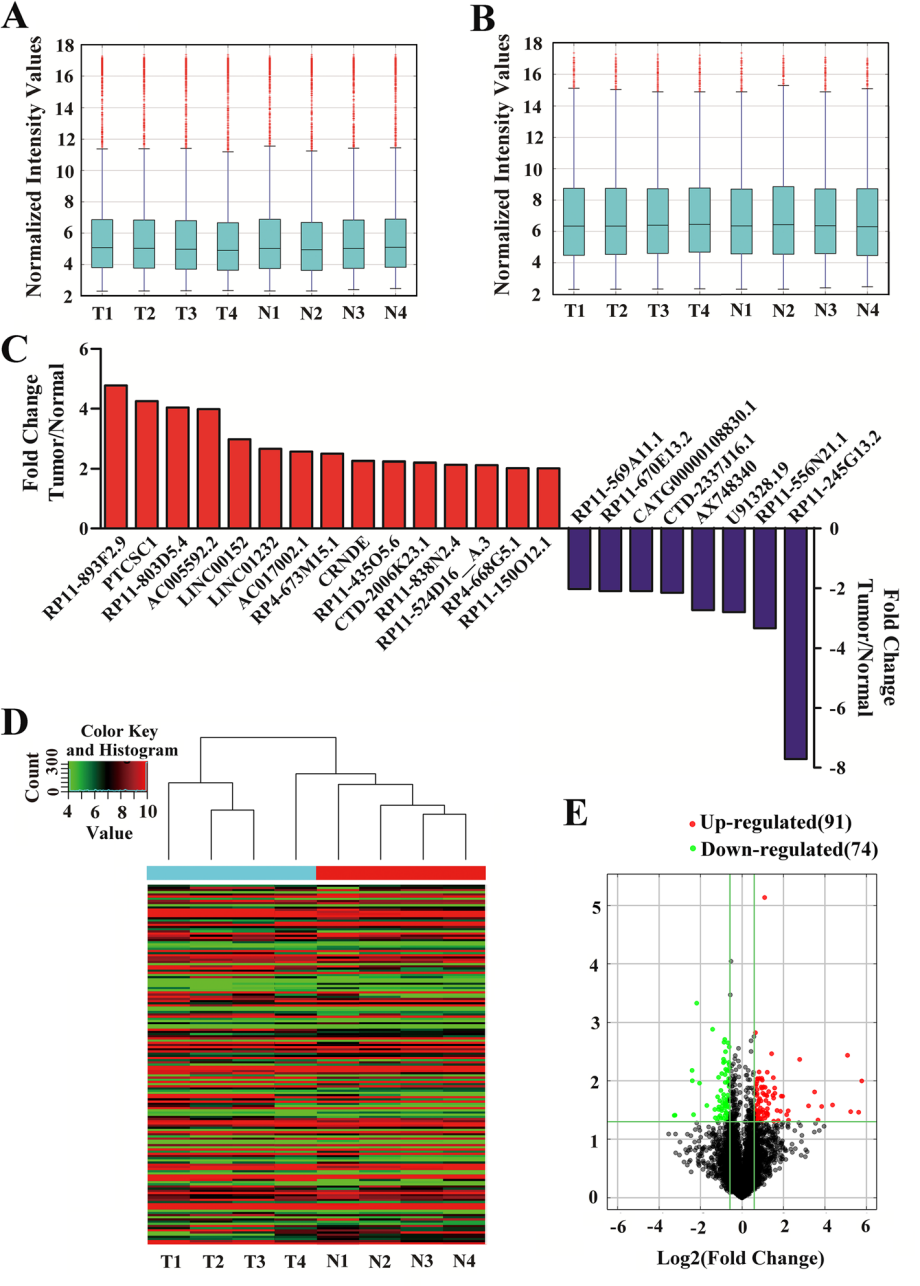
Expression profiles of SE-lncRNAs and mRNAs in CRC. **a** The distribution of SE-lncRNA signal values from microarray analysis was assessed by boxplot line diagrams. **b** The boxplot line diagrams of mRNAs. The boxplot analysis indicated that the distribution of SE-lncRNA and mRNA signal values was symmetrical and that SE-lncRNAs were expressed at lower levels than mRNAs in CRC. **c** A total of 23 differentially expressed SE-lncRNAs (15 up- and 8 downregulated SE-lncRNAs) in CRC tissues relative to peritumoral tissues (fold change > 2, *P*-value < 0.05). **d** Hierarchical cluster analysis of mRNAs that were differentially expressed (fold change > 1.5, *P*-value < 0.05) in CRC and peritumoral tissues. **e** Volcano plots visualizing the differentially expressed mRNAs. T: Tumor; N: Normal

To analyze the roles of SE-lncRNAs in CRC, we performed an SE-lncRNA microarray to profile the differentially expressed SE-lncRNAs and mRNAs in four CRC tissues and peritumoral tissues. As shown in boxplot line diagrams of SE-lncRNAs and mRNAs (Fig. 1a, b), the distribution of SE-lncRNA and mRNA signal values were properly symmetrical, and SE-lncRNAs were at lower levels than mRNAs in CRC, which is consistent with previous reports in other tissues [16]. Additionally, a total of 23 differentially expressed SE-lncRNAs were identified between the two groups: 15 up- and 8 downregulated SE-lncRNAs (fold change> 2, *P*-value < 0.05) in CRC tissues relative to peritumoral tissues (Fig. 1c). These data confirmed that the expression of SE-lncRNAs undergoes a change that cannot be ignored during CRC tumorigenesis. A total of 165 (91 up- and 74 downregulated) differentially expressed mRNAs were also identified (fold change > 1.5, *P*-value < 0.05) between the two groups (Fig. 1d, e), which will help us to search for potential target genes and further explore the biological functions of SE-lncRNAs in CRC.

### AC005592.2 is highly expressed in CRC tissues and cells

**Fig. 2:**
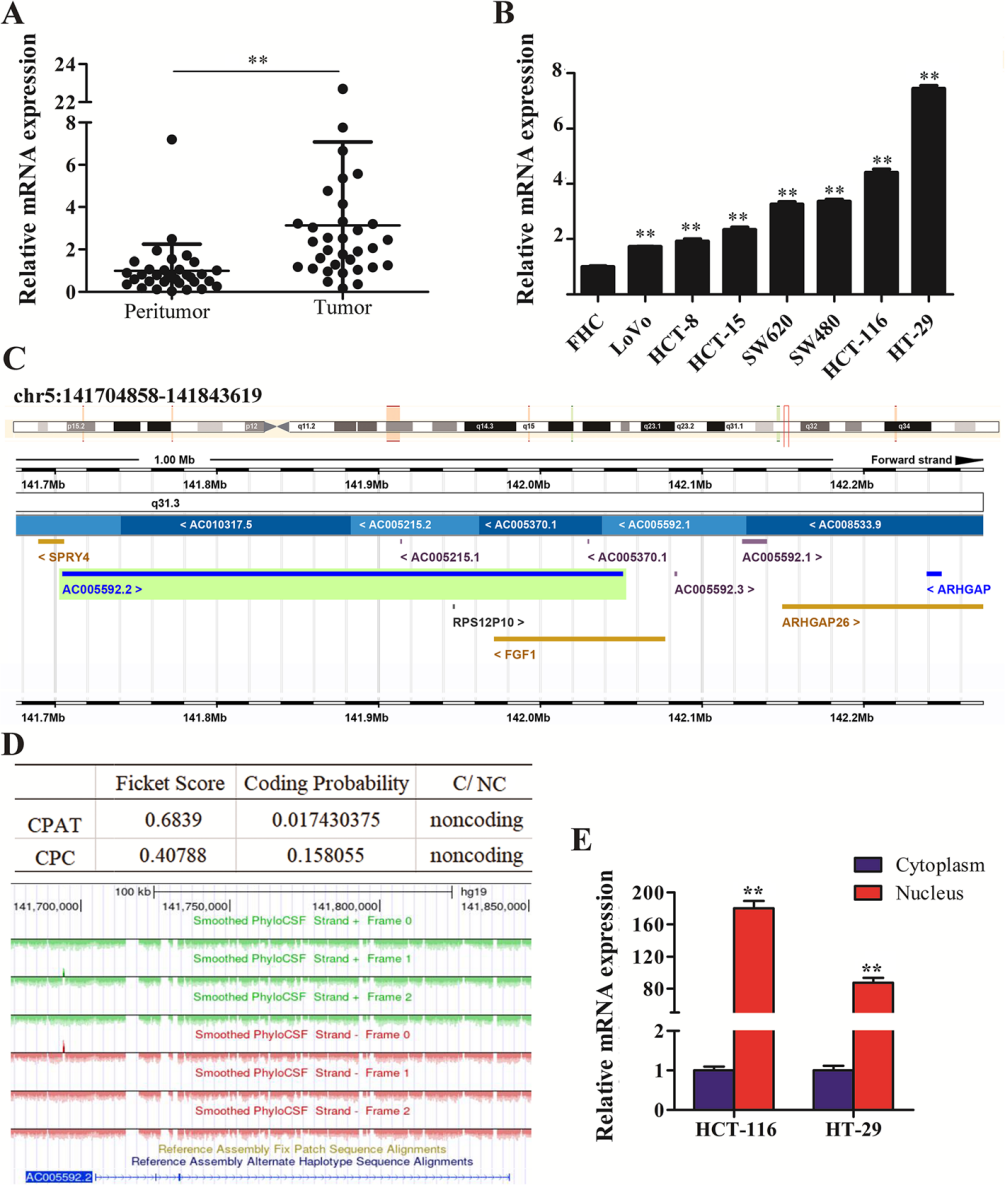
SE-lncRNA AC005592.2, which is highly expressed in CRC tissues and cells and is mostly located in the nucleus. **a** AC005592.2 expression in CRC tissues was significantly higher than that in peritumoral tissues. **b** AC005592.2 expression was higher in CRC cell lines than in the immortalized colon epithelial cell line FHC. **c** AC005592.2 is located on chr5:141,704,858-141,843,619 with a length of 591 bp as shown by the UCSC Genome Browser database. **d** AC005592.2 was predicted to be a noncoding RNA by CPC, CPAT and PhyloCSF. **e** The subcellular location of AC005592.2 expression is mostly in the nucleus. U6, nuclear control; GAPDH, cytoplasmic control. CPAT: Coding Potential Assessment Tool; CPC: Coding Potential Calculator. C/CN: Coding or noncoding. **P-value< 0.01

Among these differentially expressed SE-lncRNAs, AC005592.2 was strongly upregulated in CRC (fold change = 3.984, *P*-value = 0.022). To confirm the microarray analysis findings, we collected 33 pairs of CRC tissues, as well as paired peritumoral tissues. The qRT-PCR results showed that AC005592.2 expression in the CRC tissues was significantly higher than that in the peritumoral tissues (fold change = 3.128, *P*-value = 0.0054, Fig. 2a). Similarly, AC005592.2 expression was higher in the CRC cell lines than in the cell line FHC (Fig. 2b). The cell lines HCT-116 and HT-29 harboring highAC005592.2 expression were selected for further studies. To further assess the clinicopathological association of AC005592.2 in CRC patients, we divided the patients into two groups via the median values: the high AC005592.2 expression group (above the median) and the low AC005592.2 expression group (below the median). The patients in the high AC005592.2 expression group were more likely to have an advanced TNM stage (*P*-value = 0.037) and poor tumor differentiation (*P*-value = 0.026), but there was no significant association with other clinical parameters (Table 1).

**Table 1:** Correlations between AC005592.2 and clinicopathological characteristics in 33 CRC cases

Characteristics	AC005592.2 expression		
	Low (*n* = 16)	High (*n* = 17)	*P*-value
Age			1.000
< 60	7	7	
≥ 60	9	10	
Sex			1.000
Female	8	8	
Male	8	9	
Tumor size			0.1721
< 5 cm	11	10	
≥ 5 cm	5	7	
T stage			0.166
T1-T2	9	5	
T3-T4	7	12	
Lymph node metastasis			0.728
N0	10	9	
N1–2	6	8	
Distant metastasis			0.688
M0	13	12	
M1	3	5	
TNM stage			0.037*
I-II	12	6	
III-IV	4	11	
Tumor differentiation			0.026*
Well/moderately	14	8	
Poorly	2	9	

* *P*-value < 0.05

### AC005592.2 is a super-enhancer-associated long noncoding RNA mainly localized in the nucleus

The genomic sequence of AC005592.2(ENSG00000231185), also named SPRY4 antisense RNA 1 (SPRY4-AS1),has six transcripts, namely, ENST00000510311.1, ENST00000443800.1, ENST00000515288.1, ENST00000414314.1, ENST00000425963.1 and ENST00000514303.1, which represent distinct annotated isoforms of a single lncRNA gene. Among them, the transcript ENST00000510311.1, which mapped to chr5:141,704,858-141,843,619 with a length of 591 bp, is a CRC-related SE-lncRNA obtained from the microarray results in this study (Fig. 2c). Furthermore, AC005592.2 was classified as a noncoding RNA with coding probabilities of 0.0174 and 0.158 predicted by CPAT and C PC, respectively,as well as the resulting PhyloCSF, which indicated no evidence for AC005592.2 translation of any possible ORF (Fig. 2d). In addition, subcellular localization analysis showed that AC005592.2 is mostly located in the nucleus of CRC cells (Fig. 2e), which suggests AC005592.2 is mainly active at the transcriptional level.

### Knockdown of AC005592.2 inhibits CRC cell proliferation, invasion, and migration and induces apoptosis

**Fig. 3:**
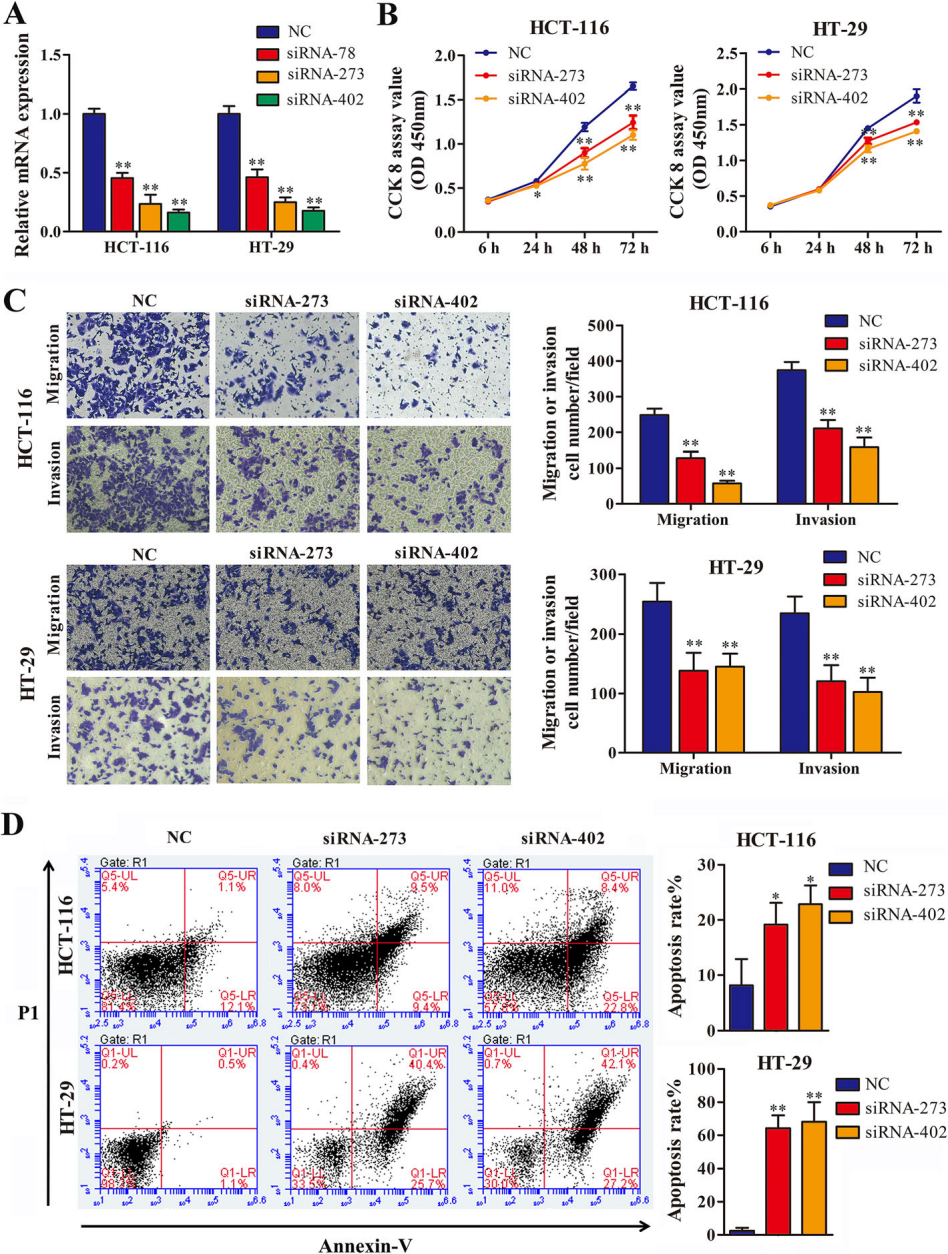
Knockdown of AC005592.2 inhibited CRC cell proliferation, invasion, and migration and induced apoptosis. **a** Relative expression of AC005592.2 in HCT-116 and HT-29 cells after transfection with AC005592.2 siRNAs. **b** CCK-8 assays revealed that AC005592.2 knockdown significantly inhibited HCT-116 and HT-29 cells proliferation. **c** Transwell assays revealed that AC005592.2 knockdown significantly inhibited migration and invasion of HCT-116 and HT-29 cells. **d** Apoptosis assays by flow cytometry indicated that AC005592.2 knockdown increased the apoptosis rate of HCT-116 and HT-29 cells. * *P*-value < 0.05, ** *P*-value < 0.01

To study the potential effects of AC005592.2 on CRC progression, we performed loss-of-function assays to evaluate the effects of AC005592.2 knockdown in CRC cell lines. Three AC005592.2 siRNAs, siRNA-402, siRNA-273 and siRNA-78, were designed to transfect HCT-116 and HT-29 cells, and the results showed that the expression level of AC005592.2 was significantly reduced following transfection with these three siRNAscompared to siRNA-NC transfection (Fig. 3a). Moreover, siRNA-402 and siRNA-273, which had good silencing efficiency, were selected for future functional assays. The CCK-8 assay showed that AC005592.2 knockdown effectively attenuated cell proliferation compared with the control (Fig. 3b). Transwell assays showed that the number of invaded and migrated cells was significantly suppressed after AC005592.2 knockdown (Fig. 3c). The flow cytometry results showed that the silencing of AC005592.2 could effectively promote cell apoptosis (Fig. 3d).

### Overexpression of AC005592.2 promotes CRC cell proliferation, invasion, and migration and inhibits apoptosis

**Fig. 4:**
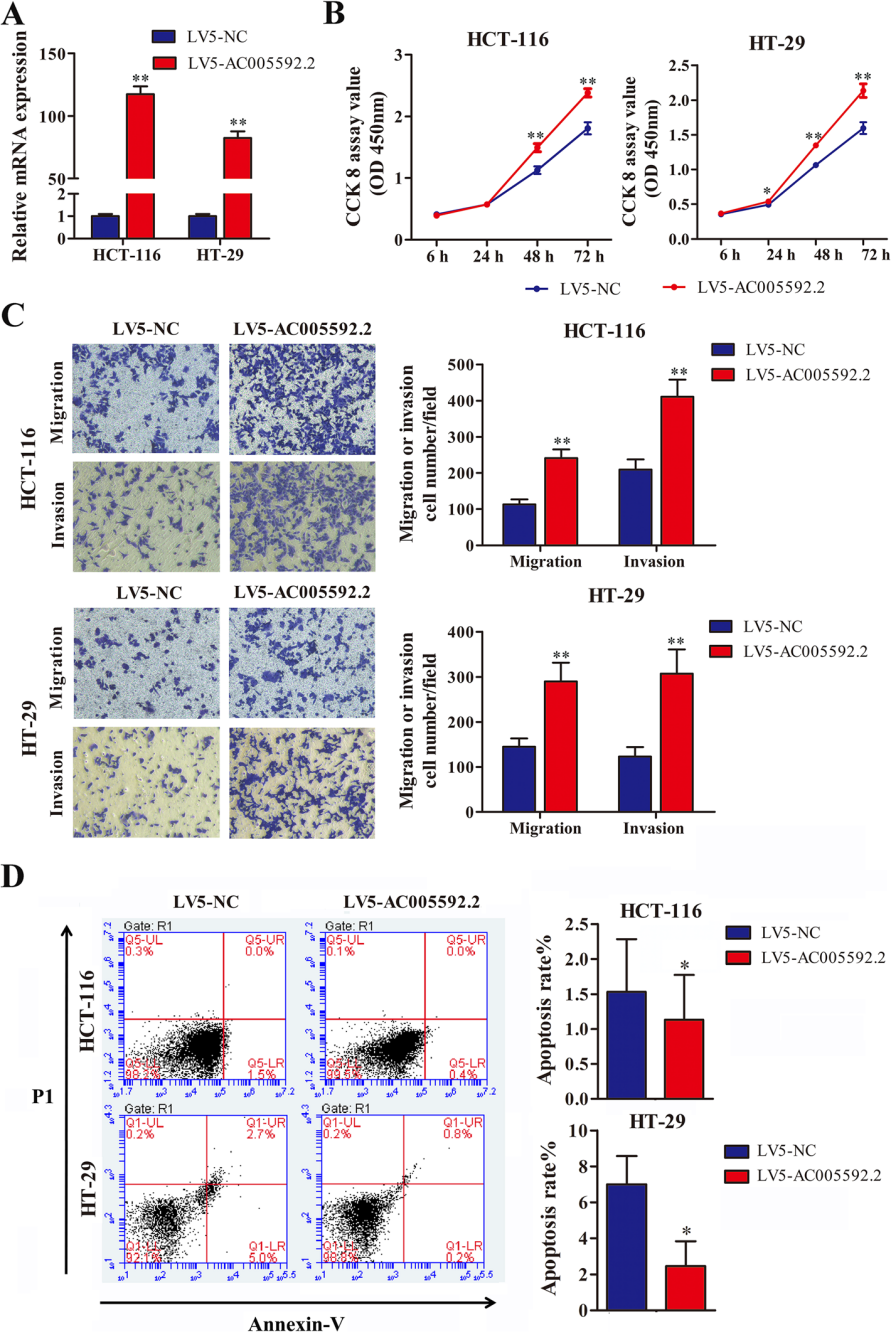
Overexpression of AC005592.2 promoted CRC cell proliferation, invasion, and migration, and inhibited apoptosis. **a** Relative expression levels of AC005592.2 in HCT-116 and HT-29 cells following LV5-NC and LV5-AC005592.2 infection**. b** CCK-8 assays revealed that AC005592.2 overexpression significantly promoted HCT-116 and HT-29 cell proliferation. **c** Transwell assays revealed that AC005592.2 overexpression significantly promoted migration and invasion of HCT-116 and HT-29 cells. **d** Apoptosis assays by flow cytometry indicated that AC005592.2 overexpression decreased the apoptosis rate of HCT-116 and HT-29 cells. * *p* < 0.05, ** *p* < 0.01

Furthermore, gain-of-function assays were used to confirm the effects of AC005592.2 overexpression in CRC cells. Compared to LV5-NC infection, LV5-AC005592.2 infection significantly increased AC005592.2 expression in HCT-116 and HT-29 cells (Fig. 4a). The functional assays showed that AC005592.2 overexpression significantly increased the proliferation of HCT-116 and HT-29 cells (Fig. 4b), while the invasive and migratory potentials of HCT-116 and HT-29 cells were effectively induced (Fig. 4c). Similarly, the overexpression of AC005592.2 in CRC cells also effectively inhibited cell apoptosis (Fig. 4d).

### The potential downstream signaling of AC005592.2

**Fig. 5:**
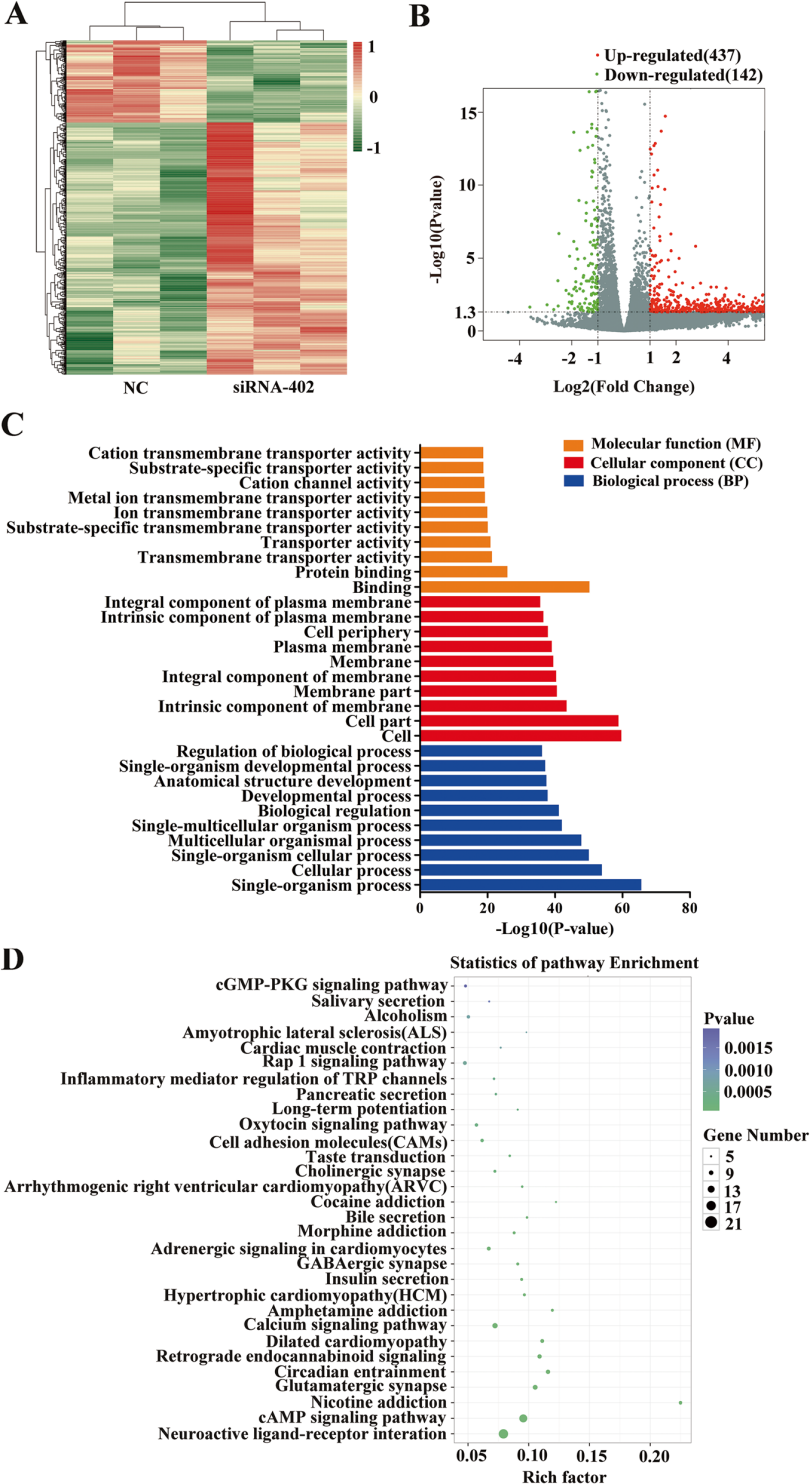
RNA-seq assays revealed potential downstream signaling of AC005592.2. **a** Hierarchical cluster analysis of dysregulated genes (fold change > 2, *P*-value < 0.05) in siRNA-treated HT-29 cells. **b** Volcano plots visualizing the dysregulated genes. **c** Gene Ontology (GO) analysis of the dysregulated genes involved in biological processes (BP), cellular components (CC) and molecular functions (MF). **d**The top 30 enriched KEGG pathways of the dysregulated genes

To explore the molecular mechanisms of AC005592.2 in promoting CRC progression, we performed RNA-seq assays to analyze gene expression changes induced by AC005592.2 silencing. Hierarchical clustering showed systematic variations in the genes between thesiRNA-402 and siRNA-NC groups (Fig. 5a). A total of 579 dysregulated genes437 up- and 142 downregulated genes were revealed following AC005592.2 knockdown, as shown by a volcano plot (fold change > 2, *P*-value < 0.05) (Fig. 5b). Furthermore, GO analysis was performed to analyze the related biological processes (BPs), cellular components (CCs) and molecular functions (MFs) of these identified genes (Fig. 5c). The AC005592.2-regulated genes were mainly involved in the following pathways: for BP: single−organism process, cellular process, and single-organism cellular process; for CC cell: cell, cell part, and intrinsic component of membrane; and for MF: binding, protein binding, and transmembrane transporter activity. Similarly, KEGG pathway enrichment analyses also revealed that AC005592.2 enrichment was associated with genes involved in neuroactive ligand-receptor interactions, the cAMP signaling pathway, nicotine addiction, and glutamatergic synapses (Fig. 5d).

### AC005592.2 directly regulates OLFM4 expression in CRC cells

**Fig. 6:**
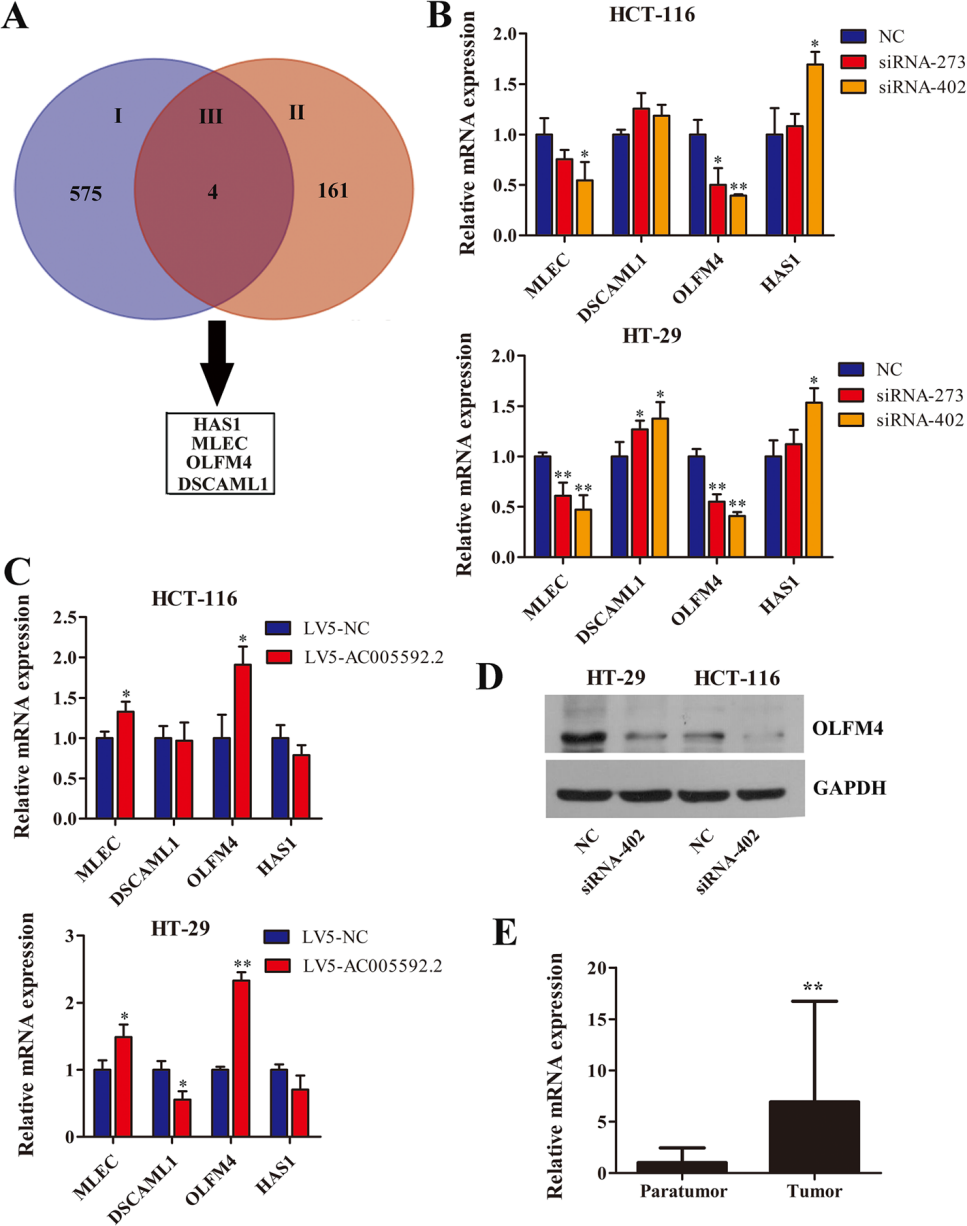
AC005592.2 directly regulates OLFM4 expression in CRC cells. **a** Four candidate genes (MLEC, DSCAML1, OLFM4, HAS1) were obtained in the study. I: 579 dysregulated genes obtained from the RNA-seq assay; II: 165 differentially expressed mRNAs identified from the SE-lncRNA microarray. III: 4 candidate genes (MLEC, DSCAML1, OLFM4, HAS1). **b** Four candidate genes were confirmed by qRT-PCR in the AC005592.2 knockdown HCT-116 and HT-29 cells. **c** Four candidate genes were confirmed by qRT-PCR in the AC005592.2-overexpressing HCT-116 and HT-29 cells. **d** OLMF4 protein levels in the AC005592.2 knockdown HCT-116 and HT-29 cells. **e** OLMF4 expression in CRC tissues was significantly higher than that in peritumoral tissues. * *P* < 0.05, ** *P* < 0.01

From the intersection of the 579 dysregulated genes with 165 differentially expressed mRNAs identified from the SE-lncRNA microarray, four candidate genes (MLEC, DSCAML1, OLFM4, HAS1) were selected (Fig. 6a). The qRT-PCR results showed that OLFM4 expression exhibited the largest fold change in HCT-116 and HT-29 cells, regardless of whether the AC005592.2 gene was knocked down or overexpressed (Fig. 6b, c). WB analysis showed that AC005592.2 downregulation significantly increased the protein expression levels of OLFM4 in HCT-116 and HT-29 cells (Fig. 6b). In addition, OLFM4 was significantly increased in the CRC tissues (fold change = 6.918, *P*-value = 0.0017, Fig. 6e), which was consistent with the data obtained from the SE-lncRNA microarray (fold change = 34.033, *P*-value = 0.0036) and RNA-seq (fold change = 2.287, *P*-value = 0.0034). These results suggested that OLFM4 is a gene downstream of AC005592.2 in CRC cells.

## Discussion

The carcinogenesis of CRC is a complex process that is generally considered to involve the activation of oncogenes or the inactivation of tumor suppressor genes [17]. Increasing evidence indicates that SE-lncRNAs are closely related to the development of multiple cancers [12], including CRC [11] and are expected to provide new therapeutic targets for CRC. An Arraystar human SE-LncRNA microarray was designed to profile lncRNAs transcribed from SE regions. With the high-performance workflow and the in-depth SE-lncRNA annotation, the microarrays produce rich lncRNA profiling data superior to RNA-seq and reveal the relationships of complex SE-lncRNA biology and regulation with some transcription factors or cancer-related genes. In this study, the SE-lncRNA microarray was used and identified a set of differentially expressed SE-lncRNAs related to CRC. Among these SE-lncRNAs, high expression of AC005592.2 in CRC tissues was positively related to TNM stage and tumor differentiation.

AC005592.2, also known as SPRY4-AS1, is located on chromosome 5q31.3 and is an antisense RNA of SPRY4. In further studies, it was found that knockdown of AC005592.2 could inhibit CRC cell proliferation, invasion and migration but promote apoptosis, while overexpression of AC005592.2 exerted the opposite effects in CRC cells. Therefore, AC005592.2 makes a crucial contribution to the carcinogenesis of CRC. Cis-regulation of the expression of adjacent genes by cisis one of the most important mechanisms of SE-lncRNAs [11, 18, 19]. We first tried to explore the molecular mechanisms of AC005592.2 in CRC by analyzing the genes that overlapped with AC005592.2 or within 50 KB of its transcription start site but without success. The basic principle of trans-acting prediction target genes is that the function of lncRNAs is related to their coexpressed protein-coding genes [20]. This study tried to predict the target gene of AC005592.2 by WGCNA coexpression analysis but still failed. Finally, RNA-seq assays were carried out to explore AC005592.2-regulated genes and pathways, and it was found that there were four genes, MLEC, DSCAML1, OLFM4, and HAS1, in both dysregulated genes obtained from RNA-seq assays and differentially expressed mRNAs identified from the SE-lncRNA microarray. Further qRT-PCR and WB analyses confirmed that OLFM4 may be a potential target of AC005592.2. Therefore, it is reasonable to believe that OLFM4 is a target gene of AC005592.2.

OLFM4, also known as hGC-1 or GW112, was first cloned from human myeloblasts. As a secreted glycoprotein, OLFM4 belongs to a family of olfactomedins and is strongly expressed in the stomach, small intestine, colon, prostate and bone marrow [21]. Previous studies have revealed that OLFM4 is closely related to several gastrointestinal malignancies, including CRC [22, 23], and its roles in the progression of CRC involve anti-inflammatory processes, proliferation, differentiation, apoptosis and cell adhesion [24]. For example, Seko N et al. examined the expression and distribution of OLFM4 in CRC by immunohistochemistry and found that34% of CRC cases were positive for OLFM4 cytoplasmic staining [25]; Liu W et al. reported that OLFM4 overexpression could alter the morphology and cortical actin distribution of HT-29 cells and decrease cell adhesion and migration [26]. In addition, the upregulation of OLFM4 was often detected in highly differentiated and early-stage CRC, while in some poorly differentiated late tumor-node-metastasis stage and metastatic CRC, downregulation or no expression was more frequently detected [26]. Interestingly, OLFM4-downregulated patients with CRChad better overall survival than OLFM4-upregulated patients [25]. Moreover, CRC progression can be attenuated by blocking the Wnt/β-catenin signaling pathway via OLFM4 negative regulation [27]. In this study, AC005592.2 positively regulated the expression of OLFM4 in CRC cells, and OLFM4 was upregulated in CRC tissues. Based on these data combined with the present work on OLFM4, wehypothesize that AC005592.2 may contribute to CRC progression by regulating OLFM4, in which multiple mechanisms might be involved.

Molecular targeted drugs with a high tumor-targeting ability and few side effects have become a research hotspot of antitumor therapies. At present, the study of targeted drugs mainly follows the principle of treating a molecule acting on one target to treat one tumor. However, the tumor is a disease characterized by multiple molecular pathological changes and various signal pathway imbalances [28, 29]. Tumor cells can adapt to new signaling pathways by self-modifying mutations, so many single-target drugs fail to show the expected effects [30–32]. Drug combinations work to a certain degree to solve this problem, but there are some limitations to this approach, such as complicated measurement design and drug interactions. The development of a single molecule that simultaneously regulates multiple mechanisms not only achieves more potent therapeutic effects but also avoids the problems caused by combined medication [33, 34]. In further investigations, many experiments will be carried out to verify that AC005592.2 contributes to CRC through multiple mechanisms, which is of considerable significance for exploring new therapies for CRC.

## Conclusions

In summary, this is the first study to systematically evaluate the role of AC005592.2 in CRC. AC005592.2 is upregulated in CRC tissues, and its overexpression may be associated with CRC progression. Therefore, these findings enable us to reasonably conclude that AC005592.2 is an oncogene in CRC and may serve as a target for new therapies in CRC, which will provide a new opportunity for CRC patients.

## Supplementary Information

**Additional file 1:.** Additional file of WBR3

**Additional file 2:.** Supplementary Table S1R3

## Abbreviations

CRC: Colorectal cancer
SE-lncRNAs: Super-enhancer-associated long noncoding
RNAs: qRT-PCR
PCR: Fluorescence quantitative real-time PCR
OLFM4: Olfactomedin 4
GAPDH: Glyceraldehyde 3-phosphate dehydrogenase
NC: Negative control
NCBI: National Center for Biotechnology Information
GEO: Gene Expression Omnibus
ATCC: American Type Culture Collection
DMEM: Dulbecco’s modified Eagle’s medium
FBS: Fetal bovine serum
SD: Standard deviation
PBS: Phosphate-buffered saline
SDS-PAGE: Sodium dodecyl sulfate-polyacrylamide gel electrophoresis
GO: Gene Ontology
KEGG: Kyoto Encyclopedia of Genes and Genomes

## References

[1] Bernardi MP, Ngan SY, Michael M, Lynch AC, Heriot AG, Ramsay RG, Phillips WA (2015). Molecular biology of anal squamous cell carcinoma: implications for future research and clinical intervention. Lancet Oncol.

[2] Khan A, Zhang X (2016). dbSUPER: a database of super-enhancers in mouse and human genome. Nucleic Acids Res.

[3] Pott S, Lieb JD (2015). What are super-enhancers?. Nat Genet.

[4] Wang X, Cairns MJ, Yan J (2019). Super-enhancers in transcriptional regulation and genome organization. Nucleic Acids Res.

[5] Thandapani P (2019). Super-enhancers in cancer. Pharmacol Ther.

[6] Yuan J, Jiang YY, Mayakonda A, Huang M, Ding LW, Lin H, Yu F, Lu Y, Loh TKS, Chow M (2017). Super-enhancers promote transcriptional Dysregulation in nasopharyngeal carcinoma. Cancer Res.

[7] Vucicevic D, Corradin O, Ntini E, Scacheri PC, Orom UA (2015). Long ncRNA expression associates with tissue-specific enhancers. Cell Cycle.

[8] Soibam B (2017). Super-lncRNAs: identification of lncRNAs that target super-enhancers via RNA:DNA:DNA triplex formation. Rna.

[9] Ounzain S, Micheletti R, Arnan C, Plaisance I, Cecchi D, Schroen B, Reverter F, Alexanian M, Gonzales C, Ng SY et al: CARMEN, a human super enhancer-associated long noncoding RNA controlling cardiac specification, differentiation and homeostasis. J Mol Cell Cardiol 2015, 89(Pt A):98–112. 10.1016/j.yjmcc.2015.09.016 26423156

[10] Hon CC, Ramilowski JA, Harshbarger J, Bertin N, Rackham OJ, Gough J, Denisenko E, Schmeier S, Poulsen TM, Severin J (2017). An atlas of human long non-coding RNAs with accurate 5′ ends. Nature.

[11] Xiang JF, Yin QF, Chen T, Zhang Y, Zhang XO, Wu Z, Zhang S, Wang HB, Ge J, Lu X (2014). Human colorectal cancer-specific CCAT1-L lncRNA regulates long-range chromatin interactions at the MYC locus. Cell Res.

[12] Guo ZW, Xie C, Li K, Zhai XM, Cai GX, Yang XX, Wu YS. SELER: a database of super-enhancer-associated lncRNA- directed transcriptional regulation in human cancers. Database The Journal of Biological Databases and Curation 2019. 2019;2019. 10.1093/database/baz027 PMC6390648 30806704

[13] Kong L, Zhang Y, Ye ZQ, Liu XQ, Zhao SQ, Wei L, Gao G: CPC: assess the protein-coding potential of transcripts using sequence features and support vector machine. Nucleic Acids Res 2007, 35(Web Server issue):W345–W349. 10.1093/nar/gkm391 PMC1933232 17631615

[14] Lin MF, Jungreis I, Kellis M (2011). PhyloCSF: a comparative genomics method to distinguish protein coding and non-coding regions. Bioinformatics.

[15] Wang Y, He L, Du Y, Zhu P, Huang G, Luo J, Yan X, Ye B, Li C, Xia P (2015). The long noncoding RNA lncTCF7 promotes self-renewal of human liver cancer stem cells through activation of Wnt signaling. Cell Stem Cell.

[16] Derrien T, Johnson R, Bussotti G, Tanzer A, Djebali S, Tilgner H, Guernec G, Martin D, Merkel A, Knowles DG (2012). The GENCODE v7 catalog of human long noncoding RNAs: analysis of their gene structure, evolution, and expression. Genome Res.

[17] Siegel RL, Miller KD, Fedewa SA, Ahnen DJ, Meester RGS, Barzi A, Jemal A (2017). Colorectal cancer statistics, 2017. CA Cancer J Clin.

[18] Anderson KM, Anderson DM, McAnally JR, Shelton JM, Bassel-Duby R, Olson EN (2016). Transcription of the non-coding RNA upperhand controls Hand2 expression and heart development. Nature.

[19] Mousavi K, Zare H, Dell'orso S, Grontved L, Gutierrez-Cruz G, Derfoul A, Hager GL, Sartorelli V (2013). eRNAs promote transcription by establishing chromatin accessibility at defined genomic loci. Mol Cell.

[20] Kopp F, Mendell JT (2018). Functional classification and experimental dissection of long noncoding RNAs. Cell.

[21] Zhang J, Liu WL, Tang DC, Chen L, Wang M, Pack SD, Zhuang Z, Rodgers GP (2002). Identification and characterization of a novel member of olfactomedin-related protein family, hGC-1, expressed during myeloid lineage development. Gene.

[22] Liu W, Rodgers GP (2016). Olfactomedin 4 expression and functions in innate immunity, inflammation, and cancer. Cancer Metastasis Rev.

[23] Quesada-Calvo F, Massot C, Bertrand V, Longuespee R, Bletard N, Somja J, Mazzucchelli G, Smargiasso N, Baiwir D, De Pauw-Gillet MC (2017). OLFM4, KNG1 and Sec24C identified by proteomics and immunohistochemistry as potential markers of early colorectal cancer stages. Clin Proteomics.

[24] Grover PK, Hardingham JE, Cummins AG (2010). Stem cell marker olfactomedin 4: critical appraisal of its characteristics and role in tumorigenesis. Cancer Metastasis Rev.

[25] Seko N, Oue N, Noguchi T, Sentani K, Sakamoto N, Hinoi T, Okajima M, Yasui W (2010). Olfactomedin 4 (GW112, hGC-1) is an independent prognostic marker for survival in patients with colorectal cancer. Exp Ther Med.

[26] Liu W, Liu Y, Zhu J, Wright E, Ding I, Rodgers GP (2008). Reduced hGC-1 protein expression is associated with malignant progression of colon carcinoma. Clin Cancer Res.

[27] Liu W, Li H, Hong SH, Piszczek GP, Chen W, Rodgers GP (2016). Olfactomedin 4 deletion induces colon adenocarcinoma in Apc (min/+) mice. Oncogene.

[28] Sonnenschein C, Soto AM (2013). The aging of the 2000 and 2011 hallmarks of Cancer reviews: a critique. J Biosci.

[29] Sonnenschein C, Soto AM (2008). Theories of carcinogenesis: an emerging perspective. Semin Cancer Biol.

[30] Haber DA, Gray NS, Baselga J (2011). The evolving war on cancer. Cell.

[31] Csermely P, Agoston V, Pongor S (2005). The efficiency of multi-target drugs: the network approach might help drug design. Trends Pharmacol Sci.

[32] Boran AD, Iyengar R (2010). Systems approaches to polypharmacology and drug discovery. Curr Opin Drug Discov Dev.

[33] Mondello P, Derenzini E, Asgari Z, Philip J, Brea EJ, Seshan V, Hendrickson RC, de Stanchina E, Scheinberg DA, Younes A (2017). Dual inhibition of histone deacetylases and phosphoinositide 3-kinase enhances therapeutic activity against B cell lymphoma. Oncotarget.

[34] Andrews FH, Singh AR, Joshi S, Smith CA, Morales GA, Garlich JR, Durden DL, Kutateladze TG (2017). Dual-activity PI3K-BRD4 inhibitor for the orthogonal inhibition of MYC to block tumor growth and metastasis. Proc Natl Acad Sci U S A.

