# Supplementary figures and images for: Super-enhancer-associated long noncoding RNA AC005592.2 promotes tumor progression by regulating OLFM4 in colorectal cancer
Source: BMC Cancer. 2021 Feb 23;21:187. doi: 10.1186/s12885-021-07900-x (PMC7903608; doi:10.1186/s12885-021-07900-x)

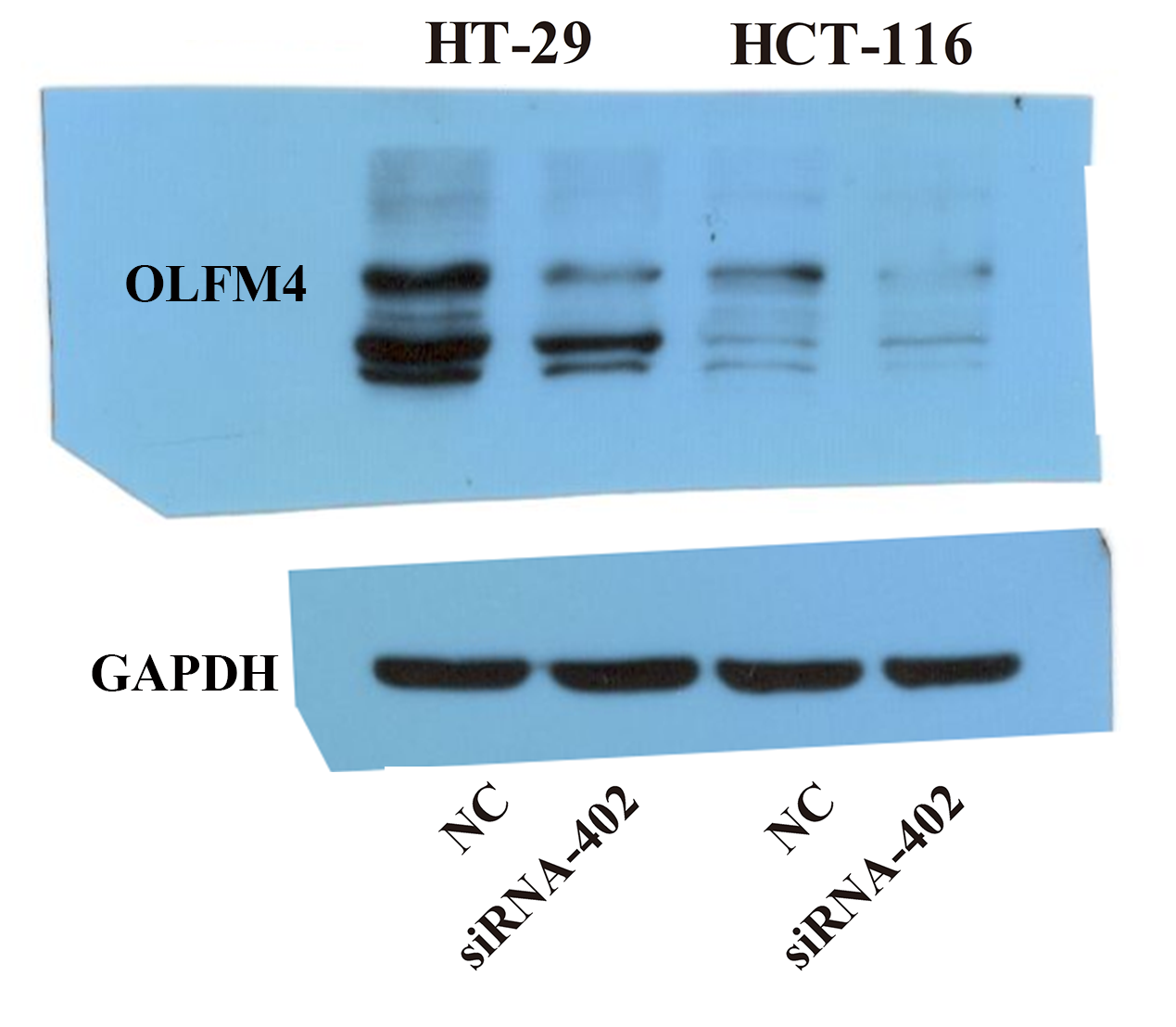

Supplement: Supplementary file 1 — Additional file 1:. Additional file of WBR3 [file 12885_2021_7900_MOESM1_ESM.tiff]
