# Supplementary material for: Super-enhancer-associated long noncoding RNA AC005592.2 promotes tumor progression by regulating OLFM4 in colorectal cancer
Source: BMC Cancer. 2021 Feb 23;21:187. doi: 10.1186/s12885-021-07900-x (PMC7903608; doi:10.1186/s12885-021-07900-x)
Supplement: Supplementary file 2 — Additional file 2:. Supplementary Table S1R3 [file 12885_2021_7900_MOESM2_ESM.docx]

**Table S1** The sequences of all primers

| Gene | Primer Sequence (5'-3') | |
| --- | --- | --- |
|  | Forward Primer | Reverse Primer |
| ACC005592.2 | AGGGAGTAGGCTGACCAGTT | TCAGCTCTTCCCGGTTGATG |
| OLFM4 | ACTGTCCGAATTGACATCATGG | TTCTGAGCTTCCACCAAAACTC |
| MLEC | CACAGTCCCAGCAAAAGGTAT | ATGCCCAACACGATCAAAGAT |
| DSCAML1 | CCCCTCCGCCTTCAATAGC | GTGTAGGGTTCCCTGAAAACTG |
| HAS1 | GAGCCTCTTCGCGTACCTG | CCTCCTGGTAGGCGGAGAT |
| GAPDH | AGAAGGCTGGGGCTCATTTG | AGGGGCCATCCACAGTCTTC |
| U6 | GCTTCGGCAGCACATATACT | GGAACGCTTCACGAATTTGC |
